# Do sociodemographic factors modify the association between antenatal care utilisation and acute respiratory infection among infants in Ethiopia?

**DOI:** 10.1371/journal.pgph.0006491

**Published:** 2026-05-14

**Authors:** Melash Asresie, Gedefaw Fekadu, Bircan Erbas, Mehak Batra

**Affiliations:** 1 School of Public Health, Bahir Dar University, Bahir Dar, Ethiopia; 2 School of Psychology and Public Health, La Trobe University, Melbourne, Victoria, Australia; University of California San Francisco, UNITED STATES OF AMERICA

## Abstract

Antenatal care (ANC) is vital for optimum maternal care and could provide an opportunity to prevent acute respiratory infection (ARI) in early childhood, particularly in low-resource settings; however, its impact on ARI remains underexplored. This study examined the association between ANC utilisation and ARI in Ethiopian infants, including stratified analyses by key socio-demographic factors. A total of 4,154 mother-infant pairs were analysed, using data from the Performance Monitoring for Action Ethiopia panel surveys conducted from 2019 to 2021 and from 2021 to 2023. At least one ANC visit, adequate ANC visits, and timely initiation of ANC were the exposures, and ARI among infants was the outcome. Generalised linear mixed models (GLMMs) were fitted using STATA 17 to estimate odds ratios (ORs) with 95% confidence intervals (CIs). Stratified analysis was conducted by maternal education, family wealth status, and administrative regions. Adequate ANC visits (≥4 ANC visits) were associated with significantly lower odds of ARI (aOR = 0.47; 95% CI: 0.24, 0.90). The protective effect was more pronounced among infants of mothers with higher education (aOR = 0.15; 95% CI: 0.04, 0.56), from wealthier households (aOR = 0.31; 95% CI: 0.14, 0.67), and those residing in agrarian regions (aOR = 0.44; 95% CI: 0.22, 0.88). At least one ANC visit showed a reduction in odds, but only among infants of educated mothers (aOR = 0.20; 95% CI: 0.05, 0.79). Adequate ANC visits significantly reduced the odds of ARI, especially among infants of educated mothers, wealthier families, and in agrarian regions. In contrast, just one visit or early initiation alone showed no benefit. Ensuring quality ANC access and targeting women in poverty with limited transport, income, and health literacy needs to be the focus of maternal and child health programs if we are to improve child health outcomes in low-resource settings like Ethiopia.

## Introduction

Despite a strong commitment from the World Health Organisation (WHO) and United Nations Children’s Fund (UNICEF), such as launching the Integrated Global Action Plan for Pneumonia and Diarrhoea (GAPPD), acute respiratory infections (ARI) remain one of the leading causes of morbidity and mortality among children under five in low- and middle-income countries (LMICs) [1]. Globally, ARI account for approximately 15% of under-five mortality [2]. While mortality from diarrhoea declined by 63% between 2000 and 2021, the reduction in pneumonia-related mortality has been slower, at 54% globally [1].

According to the 2021 Global Burden of Disease (GBD) study, the global incidence of ARI is estimated at 5,750 cases per 100,000 children per year, with substantial variation between countries, ranging from 413 in the Netherlands to 12,190 in Pakistan [3]. This wide disparity highlights the disproportionate burden of ARI in LMICs, where ARI-related mortality among children under five is strongly associated with modifiable risk factors, including malnutrition, adverse birth outcomes, limited utilisation of child and maternal healthcare services, low socioeconomic status, and poor environmental conditions [4]. Beyond mortality, ARI contribute to long-term respiratory complications and premature mortality, while also imposing a significant burden on healthcare systems, as a major cause of outpatient visits and hospitalisations [5–7]. Although effective preventive interventions such as immunisation, breastfeeding promotion, improved nutrition, and sanitation exist, utilisation and adherence to these recommendations remain limited in LMICs due to a complex interplay of low parental health literacy, cultural factors, broader socioeconomic inequality, poor access to health facilities, and under-resourced health facilities [8,9].

Globally, in 2022, only 44% of infants were exclusively breastfed; fewer than one in four received adequate complementary feeding [10], and over 21 million children were under-vaccinated in 2023 [11]. These gaps in access to services represent missed opportunities to promote behaviour change and reduce disease burden, many of which could be addressed through antenatal care (ANC).

ANC presents a platform to deliver preventive strategies aligned with the GAPPD priorities [12]. These include maternal immunisation, nutritional counselling and support, infection screening and treatment, health education, and birth preparedness. ANC may reduce infant ARI risk by equipping mothers with knowledge on hygiene, proper care, and care-seeking practices, strengthening preventive behaviours, and linking them to child health services.

However, ANC coverage remains suboptimal in LMICs. Only 60.6% of women attend four or more ANC visits, and just 11.3% complete the recommended eight contacts [13]. In Ethiopia, coverage is particularly low, with only 43% attending four visits and just 3% completing eight [14,15]. Although the World Health Organisation (WHO) now recommends a minimum of eight ANC contacts, the four-visit benchmark remains a widely used global indicator [16] and reflects the current realities of coverage in Ethiopia. Even if free maternal healthcare is available in Ethiopia, substantial inequalities persist, with over a 20% gap in ANC access between advantaged and disadvantaged women based on education, residence, and economic status [17].

Despite progress in child survival over the past two decades, Ethiopia still ranks among the countries with the highest under-five mortality rates [18]. ARI remains a major public health challenge, accounting for 17% of under-five mortality [1]. While previous studies have examined the prevalence and risk factors of ARI, limited attention has been given to how regular ANC utilisation may contribute to ARI prevention in infants [19–21]. While ANC is well recognised for improving pregnancy outcomes and reducing neonatal mortality [22], its role in preventing postnatal infections, particularly ARIs, remains underexplored.

To our knowledge, no study has specifically examined ANC as the primary exposure in relation to ARI in infants or assessed whether its impact varies by key social determinants such as maternal education or household wealth. These factors may influence how regularly women access ANC and their ability to act on preventive advice [17]. Identifying such variables can support more effective, equity-driven program design and contribute to reductions in morbidity.

We hypothesised that higher ANC utilisation would be associated with reduced odds of ARI in infants and that this association would vary by maternal education, household wealth, and administrative region.

## Methods

### Data sources and sampling procedures

This study utilised data from the Performance Monitoring for Action (PMA) Ethiopia panel surveys, a nationally representative longitudinal study designed to generate data on reproductive, maternal, and newborn health (RMNH). The data were collected by Addis Ababa University in collaboration with the Johns Hopkins Bloomberg School of Public Health (JHBSPH). To improve statistical power and precision, we combined data from two survey cohorts conducted in 2019–2021 and 2021–2023. The first cohort included participants from five regions in Ethiopia (Tigray, Afar, Amhara, Oromia, and the Southern Nations, Nationalities, and Peoples’ Region [SNNP]) and one city administration (Addis Ababa). Tigray and Afar regions were excluded from the second cohort due to conflict during data collection.

A two-stage stratified cluster sampling strategy was employed to select participants in each region. In the first stage, enumeration areas (EAs) were chosen. In the second stage, households were randomly selected within each EA (S1 Text). All pregnant women and postpartum women (0–9 weeks postpartum) aged 15–49 years residing in selected households were invited to participate in the follow-up surveys. A total of 5,154 women were enrolled on the panel, with 2,857 in cohort 1 and 2,297 in cohort 2. At enrolment, 4,036 women (78.31%) were pregnant, 568 (11.02%) were 0–4 weeks postpartum, and 550 (10.67%) were 5–9 weeks postpartum. Follow-up interviews were conducted at six weeks, six months, and 12 months postpartum, with six-week data collected concurrently at baseline for women already 5–9 weeks postpartum. Data related to ANC attendance were gathered during the six-week postpartum survey.

The present analysis focused on mother-infant pairs who participated in the six-month follow-up. A total of 4,762 mothers, between 24 and 32 weeks postpartum, were eligible across both cohorts at the six-month visit. Of these, 4,288 completed the survey, yielding a response rate of 90.05%. Among completed responses, 4,154 mothers with living children were asked about ARI symptoms in their infants within the two weeks prior to the survey (S1 Fig) [23,24].

Additional details on the PMA Ethiopia survey design, sampling methodology, and data collection tools are available online at: https://www.pmadata.org/countries/ethiopia.

### Variables included in the study

#### Outcome.

The outcome variable for this study was ARI, measured based on mothers’ recall of ARI symptoms. It was defined as a child having a cough accompanied by fast or difficult breathing in the two weeks preceding the survey. It was coded as “yes” for the presence of ARI symptoms and “no” for their absence. Asking mothers/caregivers about childhood ARI symptoms in the two weeks preceding the survey is considered a best practice and is the standard methodology for household surveys such as the Demographic and Health Surveys (DHS) [25–27].

#### Exposures.

The primary exposures were:

- Adequate ANC visits: Coded as “Yes” if the woman received at least four ANC visits, and “No” otherwise [28].
- Timely initiation of ANC: Coded as “Yes” if the first ANC visit occurred within the first trimester (≤3 months) of pregnancy, and “No” otherwise [28].
- At least one ANC visit: Coded as “Yes” if the woman had at least one ANC visit with a skilled health professional, including a doctor, nurse, health officer, midwife, or health extension worker; otherwise coded as “No” [29].

### Other variables

Confounders or adjustments were selected based on a comprehensive review of the literature on factors associated with ANC utilisation and childhood ARI (S2 Fig) [14,25,26]. The following variables were included in the analysis as covariates: maternal age (15–24, 25–34, and 35–49 years), maternal education level (no education, primary, and secondary or above), maternal marital status (in union or not in union), region (Addis Ababa, Tigray, Afar, Amhara, Oromia, and SNNP), place of residence (urban or rural), parity (0 [nulliparous], 1[primiparous], 2–4 [multiparous], and 5 or more births [grand multiparous]), and household wealth index (poor, middle, and rich), categorised following other similar studies [25,26].

### Statistical analysis

Descriptive statistics were used to summarise the distribution of maternal, household, and regional characteristics. Pairwise deletion was used for women with missing or unknown responses (S1 Text). The overall prevalence of ARI was calculated, as well as across ANC indicators and other covariates, all of which were categorised. Chi-square tests were used to assess differences between groups in ARI prevalence.

Generalised linear mixed models (GLMMs) with a logit link and random intercepts at the EAs level (the smallest geographical unit) were used to account for clustering of observations within EAs and the hierarchical structure of the data (S1 Text). Children within the cluster often share similar unmeasured characteristics, which breaches the independence assumption of ordinary logistic regression. The random intercept model accommodates this by allowing baseline log-odds of ARI to vary across clusters, which improves the validity while estimating the fixed effects. Estimated effects from these models are presented as adjusted odds ratios (aORs) with 95% confidence intervals, and statistical significance was assessed at p < 0.05. The random effect is reported as the intra-cluster community correlation coefficient (ICC), showing the percentage of variance explained by the community level [30].

A stratified analysis was conducted to explore whether the association between ANC utilisation and ARI differed by family wealth status (poor vs. rich), maternal education (lower (none or primary) vs. higher (secondary or above)), and region (city administrative [Addis Ababa] vs. agrarian regions [Tigray, Amhara, Oromia, and SNNP]), as these factors influence access to healthcare and optimal childcare practice. This approach enables examination of potential effect modification, ensuring that significant subgroup differences were not hidden by the overall estimate and helping to identify groups where ANC might have varying impacts [31]. All analyses were carried out using STATA 17.

### Ethical considerations

Permission to use the data was obtained from the Johns Hopkins Bloomberg School of Public Health (JHBSPH). Subsequently, ethics approval from La Trobe University was sought and granted with ethics number HEC25104.

## Results

Of those surveyed with data on ANC visits (n = 4,000), 78.7% of mothers (n = 3,147) reported attending at least one ANC visit, while fewer than half (42.0%, n = 1,680/3,996) reported attending adequate ANC visits (i.e., at least four ANC visits). Among those who accessed ANC, only 26.48% (n = 830/3,134) initiated care within the first trimester (S1 Table).

The overall prevalence of ARI among infants in Ethiopia was 2.2% (95% CI: 1.8, 2.7; n = 91/4,154), with notable differences based on ANC utilisation. Infants of mothers with adequate ANC visits had a significantly lower ARI prevalence (1.61%, n = 27) compared to those whose mothers had inadequate ANC visits (2.59%, n = 60; *p* = 0.035). A similar but non-significant trend was observed for infants whose mothers had at least one ANC visit compared to none (2.10% vs. 2.46%; p = 0.517; Table 1).

**Table 1:** Descriptive characteristics of participants from the PMA six-month cohort (2019-2021 and 2021-2023) in Ethiopia.

Characteristics		ARI (n = 4,154)		P-value
		No n (%)	Yes n (%)	
Maternal age				0.281
	15-24	1,356 (97.84)	30 (2.16)	
	25-34	2,097 (98.04)	42 (1.96)	
	35-49	610 (96.98)	19 (3.02)	
Maternal educational status				0.095
	No education	1,346 (97.11)	40 (2.89)	
	Primary	1,581 (98.14)	30 (1.86)	
	Secondary or above	1,136 (98.18)	21 (1.82)	
Maternal marital Status				0.527
	In-union	3,970 (97.83)	88 (2.17)	
	Not in-union	93 (96.88)	3 (3.13)	
Wealth index				0.580
	Poor	1288 (98.10)	25 (1.90)	
	Middle	631 (97.38)	17 (2.62)	
	Rich	2,144 (97.77)	49 (2.23)	
Residence				
	Urban	1,648 (97.75)	38 (2.25)	0.818
	Rural	2,415 (97.85)	53 (2.15)	
Region				0.027*
	Addis Ababa	491 (98.00)	10 (2.00)	
	Tigray	362 (99.45)	2 (0.55)	
	Afar	195 (98.48)	3 (1.52)	
	Amhara	788 (96.45)	29 (3.55)	
	Oromia	1,183 (97.77)	27 (2.23)	
	SNNP	1,044 (98.12)	20 (1.88)	
Parity (n = 4,027)				0.296
	0	676 (97.27)	19 (2.73)	
	1	1,006 (98.43)	16 (1.57)	
	2-4	1,567 (97.88)	34 (2.12)	
	5 or more	686(97.30)	19 (2.70)	
At least one ANC visit (n = 4,000)				0.517
	No	832 (97.54)	21 (2.46)	
	Yes	3,081 (97.90)	66 (2.10)	
Adequate ANC visits (n = 3,996)				0.035*
	No	2,256 (97.41)	60 (2.59)	
	Yes	1,653 (98.39)	27 (1.61)	
Timely initiation of ANC visit (n = 3,134)				0.327
	No	2,252 (97.74)	52 (2.26)	
	Yes	816 (98.31)	14 (1.69)	
Cohort year				0.005*
	Cohort 1(2019–2021)	2,295 (98.37)	38 (1.63)	
	Cohort 2 (2021–2022)	1,768 (97.09)	53 (2.93)	

**Note:** Chi-square test was used, ANC = antenatal care, ARI = Acute respiratory infection, SNNP = Southern Nations, Nationalities, and Peoples, * significant at <0.05.

In unadjusted analyses, adequate ANC visits were significantly associated with lower odds of ARI among infants (cOR = 0.56; 95% CI: 0.32, 0.97; *p* = 0.039). Timely initiation of ANC also showed a protective association, although it was not statistically significant. After adjusting for maternal age, maternal education, parity, residence, wealth index, and cohort year, a consistent protective pattern was observed across all ANC indicators. Adequate ANC visits remained significantly associated with reduced odds of ARI (aOR = 0.47; 95% CI: 0.26, 0.90; p = 0.023), indicating a 53% reduction in the odds of ARI among infants whose mothers received at least four ANC visits. However, no statistically significant associations were observed for at least one ANC visit or for timely ANC visits (Table 2). Additionally, the adjusted odds ratios for the covariates are provided in the S2 Table.

**Table 2:** Unadjusted and adjusted analyses of ANC visit on ARI among infants, based on the PMA six-month cohorts (2019-2021 and 2021-2023) in Ethiopia.

Exposure		cOR (95% CI)	aOR (95% CI)
At least one ANC visit (n = 4000/3889)			
	No	1	1
	Yes	1.08 (0.57, 2.03)	0.91 (0.48, 1.74)
Adequate ANC visits (n = 3,996/3885)			
	No	1	1
	Yes	0.56 (0.32, 0.97)*	0.47 (0.26, 0.90)*
Timely initiation of ANC visit (n = 3,134/3052)			
	No	1	1
	Yes	0.70 (0.32, 1.54)	0.74 (0.32, 1.70)

**Note**: Maternal age, maternal education, parity, residence, wealth index, and year of cohort were controlled in the adjusted analyses. 1 = reference, * significant at <0.05, n = the number of observations for crude/adjusted models.

The ICC result revealed significant clustering at the community level. For example, in the model using adequate ANC visits as the exposure, the estimated cluster-level variance was 1.84 (σ^2^ = 1.84; 95% CI: 1.01, 3.36), indicating that 36% of the variability in the odds of developing ARI was due to community-level factors (S3 Table).

### Stratification analysis by maternal education, family wealth index, and region

Significant interaction terms were observed between at least one ANC visit and maternal education (p = 0.017), as well as between adequate ANC visits and maternal education (p = 0.012; S4 Table). Stratified analysis revealed that among infants whose mothers had higher education (secondary or above), receiving at least one ANC visit and adequate ANC visits were associated with a significantly lower odds of ARI (aOR=0.22; 95% CI: 0.06, 0.80; p = 0.022; aOR=0.14; 95% CI: 0.04, 0.55; p = 0.005, respectively).

Additionally, the stratified analysis showed that among infants from rich households (those in the richest category) whose mothers received adequate ANC visits, there were 70% lower odds of ARI compared to those whose mothers received inadequate ANC visits (aOR = 0.30; 95% CI: 0.14, 0.67; p = 0.003). In contrast, no significant association was found among infants from poor households (poorer or poorest) whose mothers received adequate ANC visits. This pattern suggests a potential interaction effect between household wealth and adequate ANC utilisation (p = 0.047).

Although the interaction term between adequate ANC visits and region was not statistically significant (p = 0.184), the stratified analysis revealed that among infants in the agrarian regions, adequate ANC visits were associated with a 66% reduction in the odds of ARI (aOR=0.44, 95% CI: 0.22, 0.88, P = 0.020). No significant association was found in the city administrative region. Interaction terms between at least one ANC visit and household wealth status (p = 0.657) or region (p = 0.072) were also not statistically significant. Similarly, no significant interactions were observed between timely ANC initiation and family wealth status (p = 0.577), maternal education (p = 0.179), or region (p = 0.432). In stratified analyses, neither at least one ANC visit nor timely ANC initiation was significantly associated with ARI in any subgroup (Table 3).

**Table 3:** Stratification analysis of ANC visit on ARI by maternal education, family wealth index status, and region from the PMA six-month cohorts (2019-2021 and 2021-2023) in Ethiopia.

Exposure		Lower maternal education (primary or no education) (n = 2,847/2,844/2,133)	Higher maternal education (secondary or above) (n = 961/961/796)	Poor family (poorer or poorest) (n = 1,227/1,226/805)	Rich family (richer or richest) (n = 2,017/2,015/1,705)	Agrarian regions (n = 3,247/3,245/2,650	City administrative (457/457/359
		aOR (95% CI)	aOR (95% CI)	aOR (95% CI)	aOR (95% CI)	aOR (95% CI)	aOR (95% CI)
At least one ANC visit							
	No	1	1	1	1	1	1
	Yes	1.20 (0.60, 2.49)	0.22 (0.06, 0.80) *	0.78 (0.24, 2.60)	0.82(0.33, 2.07)	0.87 (0.44, 1.72)	3.36(0.56, 21.62)
Adequate ANC visits							
	No	1	1		1	1	1
	Yes	0.69 (0.35, 1.38)	0.14(0.04, 0.55) *	2.09 (0.68, 6.49)	0.30 (0.14, 0.67) *	0.44 (0.22, 0.88) *	1.86 (0.23, 15.36)
Timely initiation of ANC visit							
	No	1	1	1	1	1	1
	Yes	0.96 (0.37, 2.53)	0.30 (0.06, 1.15)	1.46 (0.25, 8.48)	0.70 (0.26, 1.86)	0.76 (0.31, 1.88)	0.36 (0.08, 1.73

Note: Maternal age, parity, residence, wealth index, and year of cohort in the analysis of maternal education, and maternal age, parity, residence, maternal education, and year of cohort in the analysis of family wealth index as wells maternal age, maternal education, parity, residence, wealth index, and year of cohort for region analysis were controlled. 1 = reference, * significant at <0.05, n = the number of observations for at least one ANC visit / adequate ANC visits / timely initiation of ANC visits. Agrarian regions = Tigray, Amhara, Oromia, and SNNP, City administrative = Addis Ababa.

## Discussion

This study assessed the association between ANC utilisation and ARI in infants, including subgroup analyses aligned with the global objective of reducing preventable ARI-related morbidity, particularly in low-resource settings. To our knowledge, this is the first study to examine ANC as a primary exposure in relation to childhood ARI, while also evaluating the modifying effects of key sociodemographic factors. Our findings highlight ANC as an essential preventive platform not only for improving birth outcomes but also for reducing the odds of postnatal infectious diseases. The protective effect of ANC was found to vary by maternal education, household wealth, and administrative region, reinforcing the need for equity-focused ANC strategies that should focus on poorer areas where women are likely to access ANC services and may not fully understand the implications of inadequate access.

Adequate ANC visits, defined as at least four visits during pregnancy, were significantly associated with lower odds of ARI among infants in Ethiopia. Infants whose mothers received adequate ANC had lower odds of developing ARI, underscoring the potential value of sustained engagement with maternal health services**.** While these findings suggest a protective role for ANC in preventing early childhood infections, causal inferences cannot be drawn due to the observational nature of the study. Interestingly, a study by Ahmed et al. (2024), which analysed data from 25 sub-Saharan African countries, did not find a significant association between adequate ANC visits and ARI in children under five [25]. The differing results may be partly explained by variation in child age, as our study focused exclusively on infants, while the Ahmed et al. study included a broader age range. Additionally, substantial heterogeneity in maternal education and healthcare access across countries may have influenced outcomes. Notably, 73% of mothers in the Ahmed et al. study had no formal education, compared to only 22% in ours, a difference that likely shaped ANC uptake and utilisation.

Some ANC components, like maternal and foetal assessments, are provided at every visit, while others, such as counselling on birth preparedness and child health care, are specific to later stages of pregnancy, particularly the third trimester. Attending fewer than the recommended number of visits reduces the likelihood of receiving the full range of essential services [32], thereby limiting the potential health benefits of ANC. In our study, having at least one ANC visit was not significantly associated with reduced odds of ARI in infants. While some benefit may have been anticipated, our findings are consistent with others, including facility-based research in Ethiopia, which also found no significant association between at least one ANC visit and childhood pneumonia, though these studies used a different case definition based on clinical diagnosis rather than caregiver-reported symptoms [20,21]*.* The limited benefit of a single ANC visit may reflect the reactive nature of care-seeking in many low-resource settings, where women often seek care only when unwell due to competing demands such as household chores, large families to care for and agricultural work, where health-seeking behaviours are somewhat difficult [33,34]. The effectiveness of these visits depends not only on coverage but also on the frequency, continuity, and quality of care. Variation in these dimensions across health systems is likely to contribute to inconsistent findings in the literature regarding ANC’s protective effects [35].

Our study found no statistically significant association between early ANC initiation and ARI, despite the widely recognised importance of early ANC for timely risk identification, counselling, and behavioural interventions [36]. This finding highlights the need to look beyond the timing of the first visit and focus on the continuity and quality of care across the pregnancy. Early initiation without adequate follow-up may offer limited protection, particularly if health systems are not structured to retain women in care or if subsequent visits lack the content needed to reduce infection risks [37]. These results suggest that to improve child health outcomes, health systems must strengthen not only ANC service coverage but also develop programs co-designed with women in the community to better understand their needs and how they can reduce barriers for engagement in all quality care throughout their pregnancy and beyond, maintaining retention and quality across all scheduled contacts.

Our stratified analysis revealed that the protective effect of ANC visits on ARI among infants was also modified by maternal education, household wealth status, and administrative region. Among infants of mothers with secondary or higher education, even a single ANC visit was associated with lower odds of ARI, with a stronger association observed among those who received adequate ANC visits. In contrast, no significant association was found among infants of mothers with little or no formal education. This may reflect both individual- and system-level barriers. Educated mothers may be more likely to access health information and awareness [38]. Reinforced by ANC counselling, educated mothers better understand and apply health messages, leading to informed decisions and consistent childcare [39]. Conversely, women with lower educational attainment are more likely to live in underserved settings with limited access to adequately staffed and equipped health facilities [40]. As a result, poor service quality may limit the effectiveness of ANC, even when visits occur [41].

Similarly, household wealth status also modified the effectiveness of ANC visits, with reduced odds of ARI among infants from wealthier families, and no effect detected among women from poorer households. Although ANC coverage may appear similar across socioeconomic groups, affluent mothers are more likely to use postnatal services and access higher-quality care [42]. Furthermore, as the surveys were conducted during the COVID-19 pandemic, the adoption of preventive measures, such as improved hygiene, reduced social contact, and mask use, which are more likely among more educated and wealthier families, may have contributed to lower ARI prevalence within this socioeconomic group, while the opposite trend might have been observed among lower socioeconomic status groups, potentially amplifying the difference in the effect of ANC utilisation on protecting infants from ARI across different socioeconomic strata [43]. These intersecting socioeconomic and contextual factors may have collectively masked the protective effect of ANC in low-resource settings.

The regional stratified analysis revealed a protective association between adequate ANC visits and ARI in agrarian regions, but not in the city administrative region. This may reflect differences in baseline risk and access to services. In agrarian areas, higher exposure to environmental risks, greater levels of child malnutrition, and lower baseline access to health services may make preventive messages delivered during ANC visits more impactful. In contrast, in city administrative areas, better access to health services and higher care-seeking behaviour may reduce the marginal benefit of ANC in preventing ARI [29,44,45].

This study provides a comprehensive analysis and the first evidence that the protective effect of ANC on childhood ARI risk may be influenced by additional factors, including maternal education, household wealth, and geographic context. Further studies are needed to replicate and expand upon these findings. Given the observed variation in ANC effectiveness across subgroups and evolving WHO recommendations, future research should aim to identify the optimal number and timing of ANC visits required to maximise protection against childhood infections. This would enable more targeted, equitable, and resource-efficient policy implementation across diverse settings.

A key strength of this study is the use of ANC data collected within six weeks postpartum, drawn from a nationally representative longitudinal survey and analysed using robust methods, which likely reduced recall bias compared to studies relying on retrospective reporting over more extended time frames and provided detailed insights into how ANC’s effect on childhood ARI varies across key sociodemographic factors. However, several limitations should be noted. Although the WHO now recommends a minimum of eight ANC visits, this study used a four-visit threshold because only a limited number of women met the higher benchmark. Still, four ANC visits remain a global indicator of health for women, children, and adolescents [16]. Moreover, ANC utilisation was assessed only by visit frequency and timing, so it may not reflect the full quality or content of care received; however, the consistent reduction in odds of ARI with adequate ANC visits strengthens overall confidence in the conclusion of its protective effect. The data rely on mothers’ self-reports, so social desirability bias and other differences may influence responses and how mothers interpret ARI symptoms. In addition, ARI was based on symptoms in the last two weeks; while this could reduce recall bias, it likely underestimates the prevalence of ARI that occurred over the previous six months, and the relatively small sample size may limit statistical power, particularly for subgroup analyses. Furthermore, ARI was defined based on reported cough and fast or difficult breathing, consistent with WHO guidelines; however, the inability to restrict the case definition to chest-related symptoms may have affected specificity. Despite this, the approach aligns with standard practices in population-based health surveys.

## Conclusion

Women attending adequate ANC visits were less likely to have infants experiencing ARI symptoms. The protective effect was stronger among infants of mothers with higher education, those from wealthier families and in agrarian regions. At least one ANC visit, or early initiation alone, wasn’t sufficient to demonstrate a benefit, emphasising the urgent need to improve the quality and equity of access to maternal care. Focusing on women who are less likely to attend ANC systems regularly to reach and serve delivery programs is essential for ensuring that all mothers and infants benefit equally from preventive care, especially within disadvantaged populations.

## Supporting information

S1 Text Additional details for methods and results sections. (DOCX)

S1 Fig Schematic presentation of the sampling process. (TIF)

S2 Fig Directed acyclic graph (DAG). (TIF)

S1 Table Sociodemographic characteristics of participants. (DOCX)

S2 Table Adjusted odds ratios for covariates. (DOCX)

S3 Table Random effect estimates for cluster-level variance. (DOCX)

S4 Table Interaction term p-value between exposures and key sociodemographic factors. (DOCX)
