# Supplementary material for: Do sociodemographic factors modify the association between antenatal care utilisation and acute respiratory infection among infants in Ethiopia?
Source: PLOS Glob Public Health. 2026 May 14;6(5):e0006491. doi: 10.1371/journal.pgph.0006491 (PMC13175318; doi:10.1371/journal.pgph.0006491)
Supplement: S1 Text — (DOCX) [file pgph.0006491.s001.docx]

**S1 Text**

**Additional details for the Methods and results sections**

**METHOD**

PMA Ethiopia employed a two-stage stratified cluster sampling design for its panel surveys. In the first stage, enumeration areas (EAs) were selected using probability proportional to size within defined strata: 206 EAs for Cohort 1 and 162 for Cohort 2. In Amhara, Oromia, and SNNP regions, strata were defined by both region and urban/rural classification, while in the remaining regions, strata were defined by region only. We combined the six-month dataset with the corresponding six-week and baseline datasets to ensure comprehensive maternal and infant information, as each visit dataset was supplied individually. A total of 4,154 mother-child pairs were included in the final analysis (**S1** **Fig**).

Women were eligible to take part in any survey rounds once enrolled and had given their consent. Twin second-born (Baby 2, n = 59) were excluded to avoid duplication, as statistical analysis could not account for the shared maternal ID; notably, none of the excluded twins reported ARI. The survey instrument was adapted from the Demographic and Health Survey (DHS) and earlier PMA tools, and data were collected using the Open Data Kit (ODK) platform on smartphones, enabling mobile-based, real-time data entry (1, 2). Pairwise deletion was applied; women with missing or unknown responses for adequate ANC visits (n=4) or the timing of the first ANC visit (n=13) were excluded from the respective analyses. Data related to ANC utilisation after delivery were gathered during the six-week postpartum survey. As a result, 154 women who participated only in the six-month survey, not in the six-week survey, lacked ANC data and were excluded from analyses of the corresponding ANC visits variable

**Variable selection**

We selected the variable after a comprehensive review of the literature in the area (3, 4).

Then we used a directed acyclic graph to create a minimally sufficient adjusted set to control for confounders. The minimum sufficient set included maternal age, maternal education, maternal occupation, parity, family wealth index, residence, region, media exposure, partner education, health insurance coverage, and distance to health facilities. However, maternal occupation, partner education, media exposure, health insurance coverage, and distance from health facilities were not available in the dataset (**S2** **Fig**).

**Generalised linear mixed models (GLMMs)**

We used generalised linear mixed models (GLMMs) with random intercepts at the cluster level to estimate odds ratios (ORs) and 95% confidence intervals (CIs) for the association between ANC and ARI in infants. This modelling strategy accounts for the hierarchical structure of the data, where infants are nested within clusters that often share similar socio-cultural and environmental characteristics. PMA employs a multistage cluster sampling technique. To address this, GLMMs incorporate random effects at the enumeration areas (EAs) level, allowing us to estimate fixed effects of interest (ANC) while controlling for unobserved heterogeneity among clusters. Clustering at EAs and regional levels was assessed by fitting empty (null) models with a random intercept for each level. The analysis indicated negligible variation across regions, while substantial variation was observed across EAs. Therefore, only EA-level random intercepts were retained in the final models to account for clustering.

The model is specified as: Logit(P(Yij=1)) = β₀ + β₁ANCij + β₂Xij + ... + υj

Where:

- Yij is the binary outcome (presence of ARI for infant i in cluster j)
- β₀ is the intercept
- β₁ represents the fixed effect of ANC
- Xij is the covariate vector for individual i in cluster j
- υj is the random effect for the cluster.

**Results**

Distribution of participants’ characteristics is presented in **S1 Table.**

To address cases where the timing of ANC initiation could not be determined among women with no ANC visits, we conducted a separate analysis of both the timing of ANC initiation and the frequency of ANC visits. For ANC visit frequency, we modelled both at least one ANC visit and adequate ANC visits (defined as four or more ANC visits) separately. The adjusted odds ratios for each exposure: at least one ANC visit (Model A), adequate ANC visits (Model B), and timely initiation of ANC (Model C), along with covariates, are presented below. All are adjusted for similar covariates, except for the corresponding exposure **(S2 Table)**.

Multicollinearity was assessed using VIF after fitting the fixed-effect model in Stata, and no significant multicollinearity was detected as all VIF values were below 2. All models successfully converged without warnings, and the random effect variance estimates appeared reasonable in all models. The random effect variance estimates appeared reasonable in all models (**S3 Table**).

Interaction terms were tested to assess whether the association between ANC utilisation and infant ARI was modified by key sociodemographic variables, including maternal education, wealth index, and region (**S4** **Table)**.

**References**

1. Health AAUSoP, Bill t, Population MGIf, Health RHatJHBSoP. Performance Monitoring for Action Ethiopia (PMA‐ET) Panel: Cohort 1 One‐Year Follow‐up Survey.” PMA2021/ET‐C1‐1yrFU. 2021.

2. Zimmerman L, Desta S, Yihdego M, Rogers A, Amogne A, Karp C, et al. Protocol for PMA-Ethiopia: a new data source for cross-sectional and longitudinal data of reproductive, maternal, and newborn health. Gates Open Research. 2020;4:126.

3. Ahmed KY, Dadi AF, Kibret GD, Bizuayehu HM, Hassen TA, Amsalu E, et al. Population modifiable risk factors associated with under-5 acute respiratory tract infections and diarrhoea in 25 countries in sub-Saharan Africa (2014–2021): an analysis of data from demographic and health surveys. EClinicalMedicine. 2024;68.

4. Merera AM. Determinants of acute respiratory infection among under-five children in rural Ethiopia. BMC infectious diseases. 2021;21:1-12.
