# Supplementary material for: Do sociodemographic factors modify the association between antenatal care utilisation and acute respiratory infection among infants in Ethiopia?
Source: PLOS Glob Public Health. 2026 May 14;6(5):e0006491. doi: 10.1371/journal.pgph.0006491 (PMC13175318; doi:10.1371/journal.pgph.0006491)
Supplement: S1 Table — (DOCX) [file pgph.0006491.s004.docx]

**S1 Table: Sociodemographic characteristics of participants (n=4,154) based on the PMA six-month cohort survey 2019-2021 and 2021-2023**

| Variables | | Frequency n (%) |
| --- | --- | --- |
| Maternal age | |  |
|  | 15-24 | 1,386 (33.37) |
|  | 25-34 | 2,139 (51.49) |
|  | 35-49 | 629 (15.14) |
| Maternal educational status | |  |
|  | No education | 1,386 (22.37) |
|  | Primary | 1.611 (38.78) |
|  | Secondary or above | 1,157 (27.85) |
| Maternal marital Status | | |
|  | In-union | 4,058 (9.69) |
|  | Not in-union | 96 (2.31) |
| Household wealth index | |  |
|  | Poor | 1,313 (31.61) |
|  | Middle | 648 (15.60) |
|  | Higher | 2,193 (52.79) |
| Residence |  |  |
|  | Urban | 1,686 (40.59) |
|  | Rural | 2,468 (59.41) |
| Region |  |  |
|  | Addis Ababa | 501 (12.06) |
|  | Tigray | 364 (8.76) |
|  | Afar | 198 (4.77) |
|  | Amhara | 817 (19.67) |
|  | Oromia | 1,210 (29.13) |
|  | SNNP | 1,064 (25.61) |
| Parity (n=4,027) | |  |
|  | 0 | 695 (17.28) |
|  | 1 | 1,022 (25.40) |
|  | 2-4 | 1,601 (39.80) |
|  | 5 or more | 705 (17.52) |
| At least one ANC visit (n=4,000) | | |
|  | No | 853 (21.32) |
|  | Yes | 3,147 (78.68) |
| Adequate ANC visits (n=3,996) | | |
|  | No | 2,316 (57.96) |
|  | Yes | 1,680 (42.04) |
| Timely initiation of ANC visit (n=3, 134) | | |
|  | No | 2,304 (73.52) |
|  | Yes | 830 (26.48) |
| Cohort year |  |  |
|  | Cohort 1 (2019-2021) | 2,333 (56.2) |
|  | Cohort 2 (2021-2023) | 1,821(43.8) |
