# Supplementary material for: Do sociodemographic factors modify the association between antenatal care utilisation and acute respiratory infection among infants in Ethiopia?
Source: PLOS Glob Public Health. 2026 May 14;6(5):e0006491. doi: 10.1371/journal.pgph.0006491 (PMC13175318; doi:10.1371/journal.pgph.0006491)
Supplement: S2 Table — (DOCX) [file pgph.0006491.s005.docx]

**S2 Table: Adjusted analyses of at least one ANC visit (model A), adequate ANC visits (model B), and timely initiation of ANC visits (model C) and covariates for each model on ARI among infants, based on the PMA six-month cohorts (2019-2021 and 2021-2023) in Ethiopia.**

| Variables | | **Model A** | **Model B** | **Model C** |  |
| --- | --- | --- | --- | --- | --- |
|  | | aOR (95%CI) | aOR (95%CI) | aOR (95%CI) |  |
| At least one ANC visit (n=3,889) | | |  |  |  |
|  | | No | 1 |  |  |
|  | | Yes | 0.91 (0.48, 1.74) | - | - |
| Adequate ANC visits (n=3,885) | | |  |  |  |
|  | No |  | 1 |  |  |
|  | Yes | - | 0.47 (0.26, 0.90) * | - |  |
| Timely initiation of ANC visit (n= 3052) | | |  |  |  |
|  | No |  |  | 1 |  |
|  | Yes | - | - | 0.74 (0.32, 1.70) |  |
| Maternal age | |  |  |  |  |
|  | 15-24 | 1 | 1 | 1 |  |
|  | 25-34 | 1.04 (0.63, 1.72) | 1.08 (0.66, 1.78) | 1.16 (0.64, 2.11) |  |
|  | 35-49 | 1.47 (0.64, 3.40) | 1.55 (0.67, 3.59) | 1.23 (0.44, 3.46) |  |
| Maternal educational status | |  |  |  |  |
|  | No education | 1 | 1 | 1 |  |
|  | Primary | 0.92 (0.46, 1.84) | 0.97 (0.48, 1.90) | 0.89 (0.44, 1.80) |  |
|  | Secondary or above | 0.65 (0.24, 1.71) | 0.70 (0.27, 1.82) | 0.45 (0.17, 1.17) |  |
| Wealth index | |  |  |  |  |
|  | Poor | 1 | 1 |  |  |
|  | Middle | 1.45 (0.75, 2.80) | 1.48 (0.76, 2.90) | 1.55 (0.71, 3.39) |  |
|  | Rich | 1.76 (0.62, 4.98) | 1.88 (0.68, 5.20) | 2.18 (0.69, 6.84) |  |
| Residence |  |  |  |  |  |
|  | Urban | 1 | 1 | 1 |  |
|  | Rural | 0.97 (0.35, 2.69) | 0.80 (0.30, 2.14) | 0.96 (0.33, 2.78) |  |
| Parity | |  |  |  |  |
|  | 0 | 1 | 1 | 1 |  |
|  | 1 | 0.48 (0.25, 0.91) * | 0.45 (0.23, 0.87) * | 0.45 (0.21, 0.97) * |  |
|  | 2-4 | 0.49 (0.25, 0.97) * | 0.45 (0.22, 0.90) * | 0.59 (0.27, 1.30) |  |
|  | 5 or more | 0.53 (0.19, 1.47) | 0.47 (0.17, 1.30) | 0.57 (0.14, 2.27) |  |
| Cohort year | |  |  |  |  |
|  | Cohort 1(2019-2021) | 1 | 1 | 1 |  |
|  | Cohort 2 (2021-2022) | 1.85 (1.09, 3.12) * | 1.85 (1.08, 3.16) * | 1.59 (0.86, 2.92) |  |

**Note**: Maternal age, maternal education, parity, residence, wealth index, and year of cohort were controlled in the adjusted analyses. 1= reference, * significant at <0.05, n =the number of observations for crude/adjusted models, -= The corresponding variable not included in the model.
