# Supplementary material for: Do sociodemographic factors modify the association between antenatal care utilisation and acute respiratory infection among infants in Ethiopia?
Source: PLOS Glob Public Health. 2026 May 14;6(5):e0006491. doi: 10.1371/journal.pgph.0006491 (PMC13175318; doi:10.1371/journal.pgph.0006491)
Supplement: S3 Table — (DOCX) [file pgph.0006491.s006.docx]

**S3 Table:** **Random effect estimates for cluster-level variance across three GLMMs among infants, based on the PMA six-month cohort survey of 2019-2021 and 2021-2023 in Ethiopia**

| Model (exposure variable) | Variance estimate (95 % CI) | STD errors | ICC |
| --- | --- | --- | --- |
| Model 1(at least one ANC visit) | 1.69 (0.91,3.02) | 0.53 | 0.34 |
| Model 2 (adequate ANC visits) | 1.84 (1.01,3.36) | 0.56 | 0.36 |
| Model 3 (timely initiation of ANC) | 1.40 (0.70,2.83) | 0.50 | 0.30 |
