# Supplementary material for: Do sociodemographic factors modify the association between antenatal care utilisation and acute respiratory infection among infants in Ethiopia?
Source: PLOS Glob Public Health. 2026 May 14;6(5):e0006491. doi: 10.1371/journal.pgph.0006491 (PMC13175318; doi:10.1371/journal.pgph.0006491)
Supplement: S4 Table — (DOCX) [file pgph.0006491.s007.docx]

**S4 Table: Interaction term p-value between at least one ANC visit, adequate ANC visits, or timely ANC initiation, and factors like maternal education, family wealth status, or region**.

| Variables | Interaction term P-value |
| --- | --- |
| At least one ANC visit # maternal education | 0.017 |
| Adequate ANC visits # maternal education | 0.012 |
| Timely initiation of ANC visit # maternal education | 0.179 |
| At least one ANC visit # family income | 0.657 |
| Adequate ANC visits # family income | 0.047 |
| Timely initiation of ANC visit #family income | 0.577 |
| At least one ANC visit # region | 0.072 |
| Adequate ANC visits # region | 0.184 |
| Timely initiation of ANC # region | 0.432 |

Note: “no” for at least one ANC visit, adequate ANC visit, and timely initiation of ANC, along with lower maternal education, poor family income, and city administrative status were references.
